# Right ventricular dilatation score: a new assessment to right ventricular dilatation in adult patients with repaired tetralogy of Fallot

**DOI:** 10.1186/s12872-023-03487-2

**Published:** 2023-09-14

**Authors:** Ziqin Zhou, Ying Huang, Linjiang Han, Yong Zhang, Junfei Zhao, Shusheng Wen, Jimei Chen

**Affiliations:** Department of Cardiovascular Surgery, Guangdong Cardiovascular Institute, Guangdong Provincial People’s Hospital, Guangdong Academy of Medical Sciences, Guangzhou, 510080 People’s Republic of China

**Keywords:** Tetralogy of fallot, Pulmonary valve replacement, Echocardiography, Cardiac magnetic resonance, Right ventricular dilatation

## Abstract

**Background:**

Patients with repaired tetralogy of Fallot (rTOF) experience long-term chronic pulmonary valve regurgitation resulting in right ventricular (RV) dilatation. According to current guidelines, the evaluation of patients with rTOF for RV dilatation should be based on cardiac magnetic resonance (CMR). However, for many asymptomatic patients, routine CMR is not practical. Our study aims to identify screening methods for CMR based on echocardiographic data, with the goal of establishing a more practical and cheap method of screening for severity of RV dilatation in patients with asymptomatic rTOF.

**Methods:**

Thirty two rTOF patients (mean age, 21(10.5) y, 21 males) with moderate to severe pulmonary regurgitation (PR) were prospectively recruited. Each patient received CMR and echocardiogram examination within 1 month prior to operation and collected clinical data, and then received echocardiogram examination at discharge and 3–6 months post-surgery.

**Results:**

RV moderate-severe dilatation was defined as right ventricular end-diastolic volume index (RVEDVI) ≥ 160 ml/m2 or right ventricular end-systolic volume index (RVESVI) ≥ 80 ml/m2 in 15 of 32 patients (RVEDVI, 202.15[171.51, 252.56] ml/m2, RVESVI, 111.99 [96.28, 171.74] ml/m2). The other 17 (RVESDI, 130.19 [117.91, 139.35] ml/m2, RVESVI = 67.91 [63.35, 73.11] ml/m2) were defined as right ventricle mild dilatation, i.e., RVEDVI < 160 ml/m2 and RVESVI < 80 ml/m2, and the two parameters were higher than normal values. Compared with the RV mild dilatation group, patients of RV moderate-severe dilatation have worse cardiac function before surgery (right ventricular ejection fraction, 38.92(9.19) % versus 48.31(5.53) %, *p* < 0.001; Left ventricular ejection fraction, 59.80(10.26) versus 66.41(4.15), *p* = 0.021). Patients with RV moderate-severe dilatation faced longer operation time and more blood transfusion during operation (operation time, 271.53(08.33) min versus 170.53(72.36) min, *p* < 0.01; Intraoperative blood transfusion, 200(175) ml versus 100(50) ml, *p* = 0.001). Postoperative RV moderate-severe dilatation patients have poor short-term prognosis, which was reflected in a longer postoperative hospital stay (6.59 [2.12] days versus 9.80 [5.10] days, *p* = 0.024) and a higher incidence of hypohepatia (0[0] % versus 4[26.7] %, *p* = 0.023). Patients with RV dilatation score > 2.35 were diagnosed with RV moderate-severe dilatation (AUC = 0,882; Sensitivity = 94.1%; Specificity = 77.3%).

**Conclusions:**

RV moderate-severe dilatation is associated with worse preoperative cardiac function and short-term prognosis after PVR in rTOF patients with moderate to severe PR. The RV dilatation score is an effective screening method. When RV dilatation score > 2.35, the patient is indicated for further CMR examination and treatment.

**Supplementary Information:**

The online version contains supplementary material available at 10.1186/s12872-023-03487-2.

## Introduction

Patients with tetralogy of Fallot often develop pulmonary regurgitation (PR) after initial surgical repair, which can lead to long-term chronic RV volume overload and subsequent RV dilatation, remodeling, dysfunction, and eventual middle and late-stage heart failure, arrhythmia, and sudden cardiac death [1–4]. Pulmonary valve replacement (PVR) is the current standard of therapy for these patients and can effectively improve RV dilatation and dysfunction and alleviate symptoms [5]. Accurate assessment of the severity of RV dilatation in these patients can provide guidance for subsequent PVR treatment. Currently, most studies believe that the evaluation of RV dilatation based on CMR is an important reference for PVR intervention. The guidelines set RVEDVI ≥ 160 ml/m2 or RVESVI ≥ 80 ml/m2 as the threshold for RV dilatation, which indicates that patients should undergo surgical intervention as soon as possible [6, 7]. However, CMR can be costly and time consuming, therefore, echocardiography is used more readily and routinely in screening rTOF patients. The purpose of this study was to explore a predictive score based on echocardiogram data to evaluate the degree of RV dilatation in patients more economically and conveniently, so as to provide help for the next diagnosis and treatment of such patients.

## Methods

### Patients

This prospective single-center study included patients who met the following criteria: (1) had moderate to severe PR after complete repair of tetralogy of Fallot and were scheduled for PVR, (2) were aged over 16 years, (3) had no contraindications to CMR, and (4) had no prior PVR surgery. Exclusion criteria were: (1) presence of congenital or hereditary diseases other than cardiovascular disorders, (2) malignant tumors, (3) abnormal renal function requiring dialysis, and (4) primary metabolic diseases.

### Cardiac magnetic resonance

All enrolled participants underwent a comprehensive and standardized cardiac magnetic resonance assessment. The indexed left and right ventricular volumes were determined based on the body surface area. Additionally, right ventricular ejection fraction (RVEF), CMR-derived left ventricular ejection fraction (CMR-LVEF), left ventricular cardiac output (LVCO), and right ventricular cardiac output (RVCO) were quantified as part of the evaluation.

### Echocardiography

The cardiologist performed preoperative and postoperative two-dimensional echocardiograms within 1 month prior to surgical intervention, at discharge after surgery, and at 3 months or later after surgery. The tricuspid valve function was assessed by transthoracic echocardiography, usually under the top four-chamber view. The degree of tricuspid regurgitation (TR) was evaluated using color Doppler, and the classification of TR was based on the following criteria: (1) Mild TR was defined as a ratio of the length of the reflux jet to the area of the right atrium < 20%, with a backflow jet length < 1.4 cm; (2) Moderate TR was defined as a flow rate proportion between 20%-40%, with a reflux jet length between 1.4 cm-3.0 cm; (3) Severe TR was defined as a proportion greater than 40%, with a length greater than 3.0 cm. The degree of PR was evaluated using color Doppler, and the classification of PR was based on the following criteria: (1) Mild PR is defined as the small colour flow PR jet width < 10 mm in length with a narrow origin; (2) Moderate PR is defined as the intermediate colour flow PR jet width; (3) Severe PR is defined as the large colour flow PR jet width with a wide origin; may be brief in duration.

### Surgery

PVR was performed via median thoracotomy or transcatheter intervention, with the specific procedure or concomitant surgery determined by the patient's preferences and the surgeon's judgment. Details on surgical data can be found in Table 1.

**Table 1:** Demography, preoperative characteristics and surgical information

	Overall	Right ventricle mild dilatation (*n* = 17)	Right ventricular moderate-severe dilatation (*n* = 15)	*p*
***Demography***				
Male, n (%)	21 (65.6)	10 (58.8)	11 (73.3)	0.388
Age, (y)	25.00 [19.00, 30.25]	24.00 [19.00, 29.00]	27.00 [19.50, 34.50]	0.691
High, (cm)	166.72 (8.99)	166.29 (8.64)	167.20 (9.65)	0.781
Weight, (kg)	58.00 [48.75, 68.50]	60.00 [49.00, 63.50]	56.00 [48.25, 70.25]	0.762
BSA, (m2)	1.62 [1.47, 1.73]	1.65 [1.45, 1.69]	1.59 [1.50, 1.75]	0.706
Fundamental diagnosis, n (%)				
TOF	28 (87.5)	14 (82.4)	14 (93.3)	0.274
PA with VSD	2 (6.2)	2 (11.8)	0 (0.0)	
DORV	1 (3.1)	0 (0.0)	1 (6.7)	
PA with PS	1 (3.1)	1 (5.9)	0 (0.0)	
Current diagnosis, n (%)				
Moderate-severe TR	8 (25.0)	1 (5.9)	7 (46.7)	**0.008**
Residual shunt	3 (9.4)	2 (11.8)	1 (6.7)	0.621
RVOTS	5 (15.6)	5 (29.4)	0 (0.0)	**0.022**
Age of initial repaire, (y)	5.50 [2.00, 14.50]	7.00 [2.00, 13.00]	4.00 [2.00, 16.50]	0.924
Interval between repair and PVR, (y)	16.50 [14.00, 19.25]	17.00 [13.00, 19.00]	16.00 [14.50, 20.50]	0.747
NYHA, n (%)				0.647
2	20 (62.5)	10 (58.8)	10 (66.7)	
3	12 (37.5)	7 (41.2)	5 (33.3)	
SaO2, (%)	99.00 [98.00, 100.00]	99.00 [99.00, 100.00]	99.00 [97.00, 100.00]	0.619
***Electrocardiograph***				
SR, n (%)	19 (59.4)	10 (58.8)	9 (60.0)	0.946
FVPB, n (%)	1 (3.1)	0 (0.0)	1 (6.7)	0.276
Atrial fibrillation, n (%)	2 (6.2)	1 (5.9)	1 (6.7)	0.927
Atrial flutter, n (%)	3 (9.4)	0 (0.0)	3 (20.0)	0.053
CRBBB, n (%)	29 (90.6)	15 (88.2)	14 (93.3)	0.621
QT, (ms)	398.00 [382.00, 423.50]	392.00 [382.00, 410.00]	416.00 [387.00, 448.00]	0.135
QTc, (ms)	444.50 [424.50, 462.00]	439.00 [423.00, 447.00]	465.00 [432.00, 485.00]	**0.036**
QRS duration, (ms)	168.00 [134.00, 178.50]	156.00 [112.00, 172.00]	170.00 [162.00, 184.00]	0.162
QRS axis	72.50 (77.94)	80.24 (59.17)	63.73 (96.41)	0.559
PR interval, (ms)	163.00 [140.50, 178.50]	164.00 [149.00, 178.00]	162.00 [132.00, 175.00]	0.298
***Laboratory examination***				
TP, (g/L)	72.95 [67.77, 75.88]	74.90 [72.90, 78.70]	68.20 [66.80, 72.50]	**0.018**
Tbil, (umol/L)	15.30 [11.85, 20.58]	15.30 [13.20, 19.70]	15.40 [10.65, 23.10]	0.97
Cbil, (umol/L)	3.15 [2.45, 4.18]	3.10 [2.70, 3.80]	3.20 [2.15, 4.60]	0.985
ALT, (U/L)	18.50 [15.50, 31.50]	21.00 [18.00, 35.00]	17.00 [14.50, 23.00]	0.316
LDH, (U/L)	162.00 [136.75, 177.25]	171.00 [161.00, 185.00]	143.00 [131.50, 162.00]	**0.015**
N-BNP, (pg/ml)	108.30 [72.65, 205.68]	91.80 [59.20, 111.30]	236.50 [90.65, 419.45]	**0.01**
TnT, (pg/ml)	6.45 [5.00, 7.70]	5.90 [5.00, 7.60]	6.90 [5.00, 9.45]	0.478
***Surgical information***				
Type of PVR				**0.02**
Transcatheter PVR, n (%)	25 (78.1)	16 (94.1)	9 (60.0)	
Surgical PVR, n (%)	7 (21.9)	1 (5.9)	6 (40.0)	
Time of operation, (min)	217.88 (103.08)	170.53 (72.36)	271.53 (108.33)	**0.004**
CPB time, (min)	0.00 [0.00, 64.50]	0.00 [0.00, 0.00]	45.00 [0.00, 161.00]	**0.005**
ACC time, (min)	0.00 [0.00, 0.00]	0.00 [0.00, 0.00]	0.00 [0.00, 14.00]	0.113
Concomitant procedures, n (%)				
TVP	7 (21.9)	1 (5.9)	6 (40.0)	**0.02**
MVP	1 (3.1)	1 (5.9)	0 (0.0)	0.34
RVOT muscle resection	3 (9.4)	1 (5.9)	2 (13.3)	0.471
TBDP	3 (9.4)	2 (11.8)	1 (6.7)	0.621
VSD closure	1 (3.1)	0 (0.0)	1 (6.7)	0.279
Pulmonary trunk repair	2 (6.2)	1 (5.9)	1 (6.7)	0.927
PAB	2 (6.2)	1 (5.9)	1 (6.7)	0.927
Bleeding in operation, (ml)	100.00 [72.50, 200.00]	100.00 [50.00, 100.00]	200.00 [125.00, 300.00]	**0.001**

Data are count (%), mean (SD), or median [25th–75th percentiles]; *p* values < 0.05 are shown in bold

*BSA* Body surface area, *TOF* Tetralogy of fallot, *PA* Pulmonary atresia, *VSD* Ventricular septal defect, *DORV* Double outlet right ventricle, *PS* Pulmonary stenosis, *RVOTS* Right ventricular outflow tract stenosis, *PR* Pulmonary regurgitation, *TR* Tricuspid regurgitation, *PVR* Pulmonary valve replacement, *NYHA* New York Heart Association, *SaO2* pulse oxygen saturation, *SR* Sinus rhythm, *FVPB* Frequent ventricular premature beat, *CRBBB* Complete right bundle branch block, *Tbil* Total bilirubin, *Cbil* Conjugated bilirubin, *ALT* Alanine aminotransferase, *LDH* Lactic dehydrogenase, *N-BNP* N-terminal pro-B-type natriuretic peptide, *TnT* Hypersensitive troponin, *CPB* Cardiopulmonary bypass, *ACC* Aortic cross clamp, *TVP* Tricuspid valve plasty, *MVP* Mitral valve plasty, *TBDP* Transcatheter balloon dilatation plasty, *PAB* Pulmonary artery banding

### Statistical analysis

Demographic, medical history, echocardiography, CMR, and follow-up data were collected and analyzed in this study. Patients were grouped according to the latest American Heart Association guidelines based on RV dilatation: RV moderate-severe dilatation group (RVEDVI ≥ 160 ml/m2 or RVESVI ≥ 80 ml/m2) and RV mild dilatation group (RVEDVI < 160 ml/m2 and RVESVI < 80 ml/m2) [7]. RVEDVI and RVESVI of patients in RV mild dilatation group should be higher than normal [8]. The frequency (percentage) of classified data was presented. For data conforming to normal distribution, mean (standard deviation) was used, while for data not conforming to normal distribution, median [25th percentile, 95th percentile] was used. Statistical tests such as Student’s t test or paired t test, Wilcoxon signed rank test, Chi-square test or McNamar-Bowker test were used as appropriate. Correlations among continuous variables were evaluated by Pearson or Spearman correlation analysis. Relevant variables were screened by LASSO regression to establish a scoring model, and the optimal cut-off value was selected based on the Youden index from the ROC curve. Significance was set at *P* < 0.05 on both sides. The statistical analysis software used were R, version 4.0, and Spss, version 24.

## Results

### Patient characteristics

32 rTOF patients were prospectively recruited (age, 25.00[19.00,30.25] years; 21 males). Of these, 15 patients (age, 27 [19.50, 34.50] years,11 males) were classified as the RV moderate-severe dilatation group, while 17 patients (age,24.00 [19.00, 29.00] in the RV mild dilatation group; 10 males) were classified as RV mild-dilatation group. Additional baseline data and surgical information are shown in Table 1.

The proportion of patients with moderate and severe TR (7 [46.7] % versus 1[5.9] %, *p* = 0.008) in RV moderate-severe dilatation group was significantly higher than that in RV mild dilatation group. On the contrary, the proportion of right ventricular outflow tract stenosis (0 [0.0] % versus 5 [29.4] %, *p* = 0.022) was significantly lower than that of RV mild dilatation group. Laboratory examination results showed that the levels of total protein (TP) (68.20 [66.80, 72.50] versus 74.90 [72.90, 78.70], *p* = 0.018) and lactate dehydrogenase (LDH) ( 143.00 [131.50, 162.00] versus 171.00 [161.00, 185.00], *p* = 0.015) in peripheral blood of RV moderate-severe dilatation group were significantly lower than those of the other group, but the level of N-terminal B-type natriuretic peptide (N-BNP) (236.50 [90.65, 419.45] versus 91.80 [59.20, 111.30], *p* = 0.01) in peripheral blood was significantly higher than that of RV mild dilatation group (Table 1).

### Preoperative CMR and echocardiograph data

The patient underwent CMR and echocardiography less than 1 month before surgery. According to the CMR data, patients with RV moderate-severe dilatation had significantly lower REVF (38.92 [9.19] % versus 48.31 [5.53] %, *p* = 0.001), RVEDVI (202.15 [171.51, 252.56] ml/m2 versus 130.19 [117.91, 139.35] ml/m2, *p* < 0.001), and RVESVI (111.99 [96.28, 171.74] ml/m2 versus 67.91 [63.35, 73.11] ml/m2, *p* < 0.001) compared to those in the RV mild dilatation group. However, no significant differences were observed in other measurements, including PR fraction, CME-LVEF, LVCO, and RVCO (Table 2). Echocardiogram data revealed that patients in the RV moderate-severe dilatation group had significantly reduced RVOT blood flow velocity (0.76 [0.62, 0.88] m/s versus 1.00 [0.75, 1.40] m/s, *p* = 0.043), tissue doppler tricuspid annulus systolic velocity (S’) (6.69 [1.26] cm/s versus 8.01 [1.24] cm/s, *p* = 0.006), and LVEF (59.80 [10.26] % versus 66.41 [4.15] %, *p* = 0.021). In contrast, the right atrial superior and inferior diameter (RASID) index (38.93 [34.37, 42.28] mm/m2 versus 32.76 [30.42, 35.14] mm/m2), main pulmonary artery (MPA) diameter (28.27 [5.46] mm versus 22.92 [3.24] mm, *p* = 0.002), and left pulmonary artery (LPA) diameter (14.40 [10.55, 16.90] mm versus 10.90 [9.60, 12.20] mm, *p* = 0.033) were significantly increased in patients with RV moderate-severe dilatation. Among the 32 patients, 29 had TR to some extent before surgery, and patients in the RV moderate-severe dilatation group had significantly more moderate and severe tricuspid regurgitation (73.3%) compared to those in the RV mild dilatation group (11.8%) (Table 3).

**Table 2:** Preoperative CMR data

	Overall	Right ventricle mild dilatation (*n* = 17)	Right ventricular moderate-severe dilatation (*n* = 15)	*p*
RVEDVI, (ml/m2)	150.58 [127.82, 200.46]	130.19 [117.91, 139.35]	202.15 [171.51, 252.56]	** < 0.001**
RVESVI, (ml/m2)	77.85 [67.34, 109.83]	67.91 [63.35, 73.11]	111.99 [96.28, 171.74]	** < 0.001**
PR fraction, (%)	63.53 (17.38)	60.04 (13.87)	67.49 (20.43)	0.232
CMR-LVEF, (%)	57.55 [51.29, 61.84]	58.74 [55.51, 61.79]	51.44 [43.98, 60.94]	0.073
RVEF, (%)	43.91 (8.75)	48.31 (5.53)	38.92 (9.19)	**0.001**
LVCO, (L/min)	5.44 (1.52)	5.26 (1.10)	5.63 (1.91)	0.509
RVCO, (L/min)	8.24 [6.84, 9.43]	7.00 [6.60, 8.80]	9.05 [7.28, 10.82]	0.059

Data are count (%), mean (SD), or median [25th–75th percentiles]; *p* values < 0.05 are shown in bold

*CMR* Cardiovascular magnetic resonance, *RVEDVI*, Right ventricular end-diastolic volume index, *RVESVI* Right ventricular end-systolic volume index, *PR* Pulmonary regurgitation, *LVEF* Left ventricular ejection fraction, *RVEF* Right ventricular ejection fraction, *LVCO* Left ventricular cardiac output, *RVCO* Right ventricular cardiac output

**Table 3:** Preoperative echocardiograph data

	Overall (*n* = 32)	Normal right ventricle (*n* = 17)	Right ventricular moderate-severe dilatation (*n* = 15)	*p*
AO diameter, (mm)	29.00 [27.75, 31.00]	29.00 [27.00, 31.00]	29.00 [28.50, 30.50]	0.939
LA diameter, (mm)	32.00 [29.00, 36.00]	32.00 [29.00, 33.00]	32.00 [29.50, 38.50]	0.494
RVOT anteroposterior diameter index, (mm)	20.06 [18.18, 21.47]	18.90 [16.95, 21.44]	20.55 [19.76, 21.38]	0.162
RVOT gradient, (mmHg)	3.00 [2.00, 8.00]	4.00 [2.00, 8.00]	3.00 [1.75, 5.50]	0.2
RVOT blood flow velocity, (m/s)	0.82 [0.68, 1.12]	1.00 [0.75, 1.40]	0.76 [0.62, 0.88]	**0.043**
RASID index, (mm/m2)	34.37 [32.45, 38.38]	32.76 [30.42, 35.14]	38.93 [34.37, 42.28]	**0.001**
RVSID index, (mm/m2)	39.11 (5.12)	37.56 (4.30)	40.87 (5.54)	0.067
MPA diameter, (mm)	25.42 (5.12)	22.92 (3.24)	28.27 (5.46)	**0.002**
LPA diameter, (mm)	11.65 [9.90, 14.55]	10.90 [9.60, 12.20]	14.40 [10.55, 16.90]	**0.033**
RPA, (mm)	12.56 (2.46)	11.91 (2.09)	13.29 (2.70)	0.112
PVD, (mm)	22.93 (4.37)	22.45 (3.60)	23.48 (5.19)	0.514
IVC, (mm)	17.02 (4.89)	15.63 (4.69)	18.59 (4.78)	0.087
IVST, (mm)	9.16 (1.74)	9.01 (1.28)	9.33 (2.19)	0.618
RVED anteroposterior diameter, (mm)	19.21 [18.03, 22.04]	18.90 [18.03, 21.44]	20.46 [18.06, 26.18]	0.264
E/e', (m/s)	10.32 (3.36)	10.59 (3.16)	10.00 (3.66)	0.626
E, (m/s)	0.58 (0.11)	0.58 (0.10)	0.58 (0.11)	0.94
E', (cm/s)	9.08 (2.44)	9.06 (2.47)	9.11 (2.50)	0.962
FAC, (%)	42.85 (8.93)	46.12 (5.81)	39.15 (10.50)	**0.025**
TAPSE, (mm)	16.40 [15.83, 17.45]	16.40 [15.90, 17.00]	16.50 [15.75, 18.25]	0.985
S', (cm/s)	7.39 (1.40)	8.01 (1.24)	6.69 (1.26)	**0.006**
e', (cm/s)	9.08 (2.44)	9.06 (2.47)	9.11 (2.50)	0.962
RVSP, (mmHg)	32.50 [24.75, 37.00]	31.00 [25.00, 37.00]	33.00 [25.00, 35.00]	0.940
LVEF	63.31 (8.22)	66.41 (4.15)	59.80 (10.26)	**0.021**
TR, n (%)				**0.003**
no	4 (12.5)	3 (17.6)	1 (6.7)	
mild	15 (46.9)	12 (70.6)	3 (20.0)	
moderate	7 (21.9)	2 (11.8)	5 (33.3)	
severe	6 (18.8)	0 (0.0)	6 (40.0)	
PR area, (cm2)	11.21 (3.17)	11.24 (3.28)	11.19 (3.17)	0.966
PV peak gradient, (mmHg)	13.00 [7.00, 19.00]	13.00 [11.00, 18.00]	9.00 [6.15, 20.00]	0.272
PV mean gradient, (mmHg)	16.09 (7.99)	14.29 (7.09)	18.13 (8.69)	0.179
PV velocity, (m/s)	1.80 [1.43, 2.18]	1.87 [1.78, 2.09]	1.50 [1.15, 2.40]	0.234
PS, n (%)	10 (31.2)	7 (41.1)	3 (20)	0.356

Data are count (%), mean (SD), or median [25th–75th percentiles]; *p* values less than 0.05 are shown in bold

*AO* Aod aortic, *LA* Left atrial, *RVOT* Right ventricular outflow tract, *RASID* Right atrial superior and inferior diameter, *RVSID* Right ventricular superior and inferior diameter, *MPA* Main pulmonary artery, *LPA* Left pulmonary artery, *RPA* Right pulmonary artery, *PVD* Pulmonary valve diameter, *IVC* Inferior vena cava, *IVST* Interventricular septal thickness, *RVED* Right ventricular end diastolic, *E/E'* Early diastolic velocity of the tricuspid valve/early diastolic velocity of the lateral wall of the tricuspid annulus, *E* Early peak diastolic flow rate of tricuspid valve, *E*’ Doppler flow velocity of tricuspid annulus tissue during diastole, *FAC* Fractional area change, *TAPSE* Tricuspid annular plane systolic excursion, *S*’ Tissue Doppler tricuspid annulus systolic velocity, *e*’ Tissue Doppler tricuspid annulus diastolic velocity, *RVSP* Right ventricular systolic pressure, *LVEF* Left ventricular ejection fraction, *TR* Tricuspid regurgitation, *PR* Pulmonary regurgitation, *PV* Pulmonary valve, *PS* Pulmonary stenosis

### Correlation between preoperative CMR and echocardiograph

**Fig. 1:**
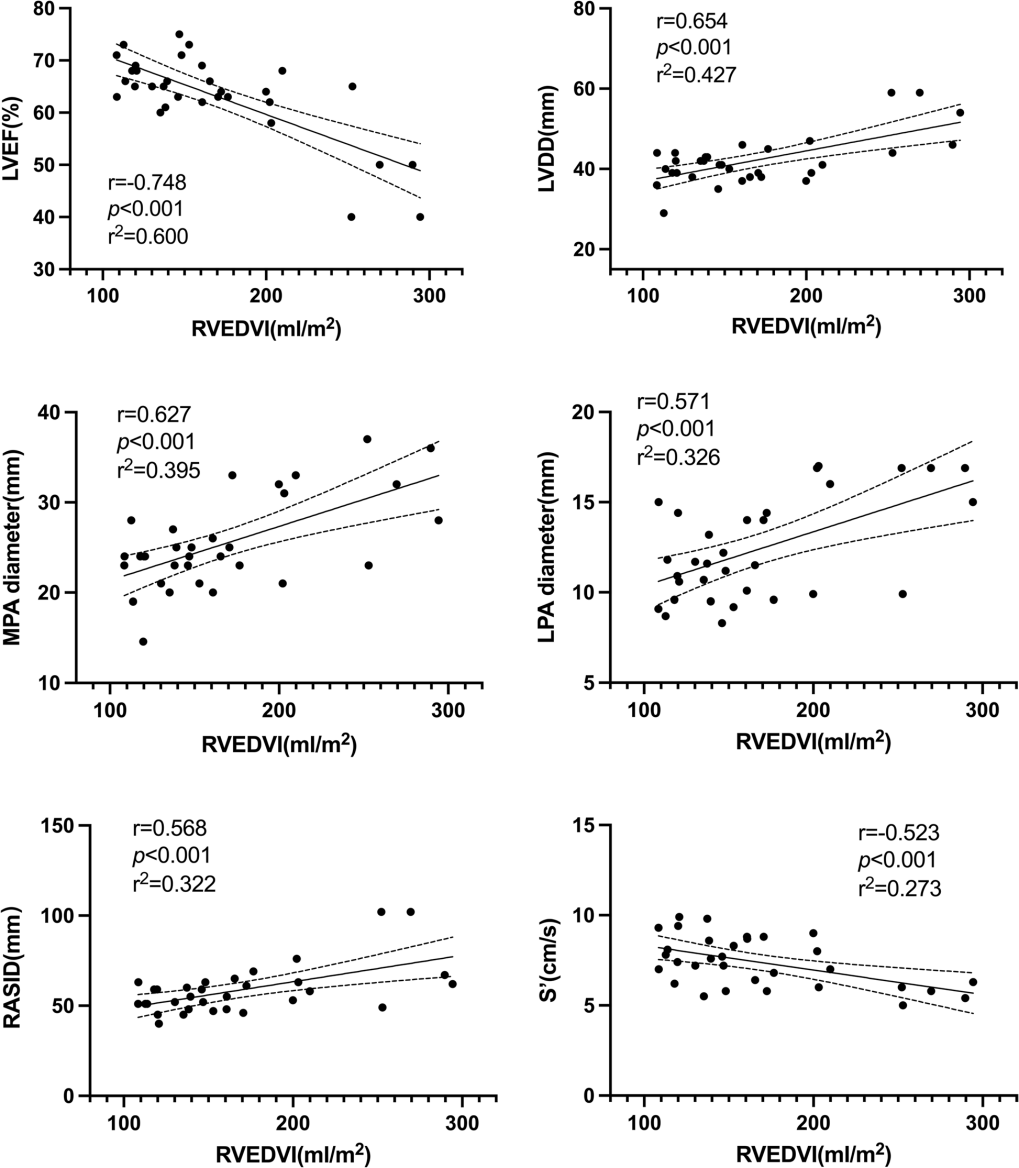
Linear correlation analysis between different echocardiographic parameters and RVEDVI. *RVEDVI* Right ventricular end-diastolic volume index, *LVEF* Left ventricular ejection fraction, *LVDD* Left ventricular diastolic diameter, *MPA* Main pulmonary artery, *LPA* Left pulmonary artery, *RASID* Right atrial superior and inferior diameter, *S*’ Tissue doppler tricuspid annulus systolic velocity

**Fig. 2:**
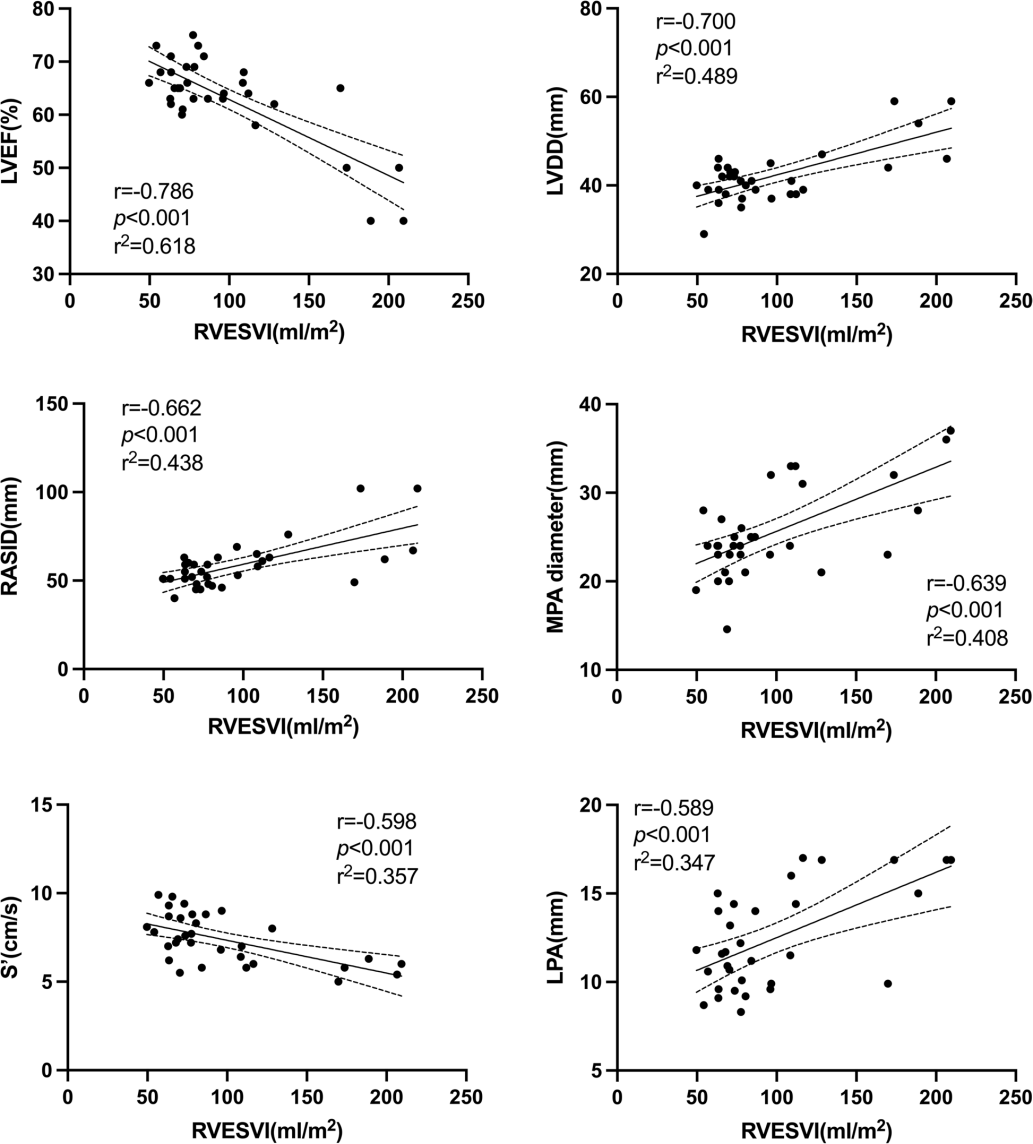
Linear correlation analysis between different echocardiographic parameters and RVESVI. *RVESVI* Right ventricular end-systolic volume index, *LVEF* Left ventricular ejection fraction, *LVDD* Left ventricular diastolic diameter, *MPA* Main pulmonary artery, *LPA* Left pulmonary artery, *RASID* Right atrial superior and inferior diameter, *S*’, tissue doppler tricuspid annulus systolic velocity

We conducted correlation analyses between echocardiograph parameters and CMR parameters that showed significant differences between the two groups, and the results are presented in Table S1 and Figure S1. The correlation analyses revealed that RVEDVI and LVEF (*r *= -0.748, *p* < 0.01), left ventricular diastolic diameter(LVDD) (*r* = 0.654, *p* < 0.001), MPA diameter (*r* = 0.627, *p* < 0.001), LPA diameter (*r* = 0.571, *p* < 0.001), RASID (*r* = 0.568, *p* < 0.001), and tissue Doppler tricuspid annulus systolic velocity(S’) (*r* = -0.523, *p* < 0.001) were significantly correlated (Table S1, Fig. 1). Similarly, RVESVI and LVEF (*r* = -0.786, *p* < 0.001), LVDD (*r* = -0.700, *p* < 0.001), MPA diameter (*r* = 0.627, *p* < 0.001), LPA diameter (*r* = -0.589, *p* < 0.001), RASID (*r* = -0.662, *p* < 0.001), and S’ (*r* = -0.598, *p* < 0.001) were significantly correlated (Table S1, Fig. 2). Moreover, RVEF and RASID (*r* = -0.651, *p* < 0.001), FAC (*r* = 0.618, *p* < 0.001), S’ (*r* = 0.594, *p* < 0.001), LVEF (*r* = 0.593, *p* < 0.001), LVDD (*r* = -0.567, *p* < 0.001), and RVSID (-0.528, *p* = 0.002) were significantly correlated (Table S1, Figure S2).

### Postoperative echocardiograph and short-term follow-up

**Fig. 3:**
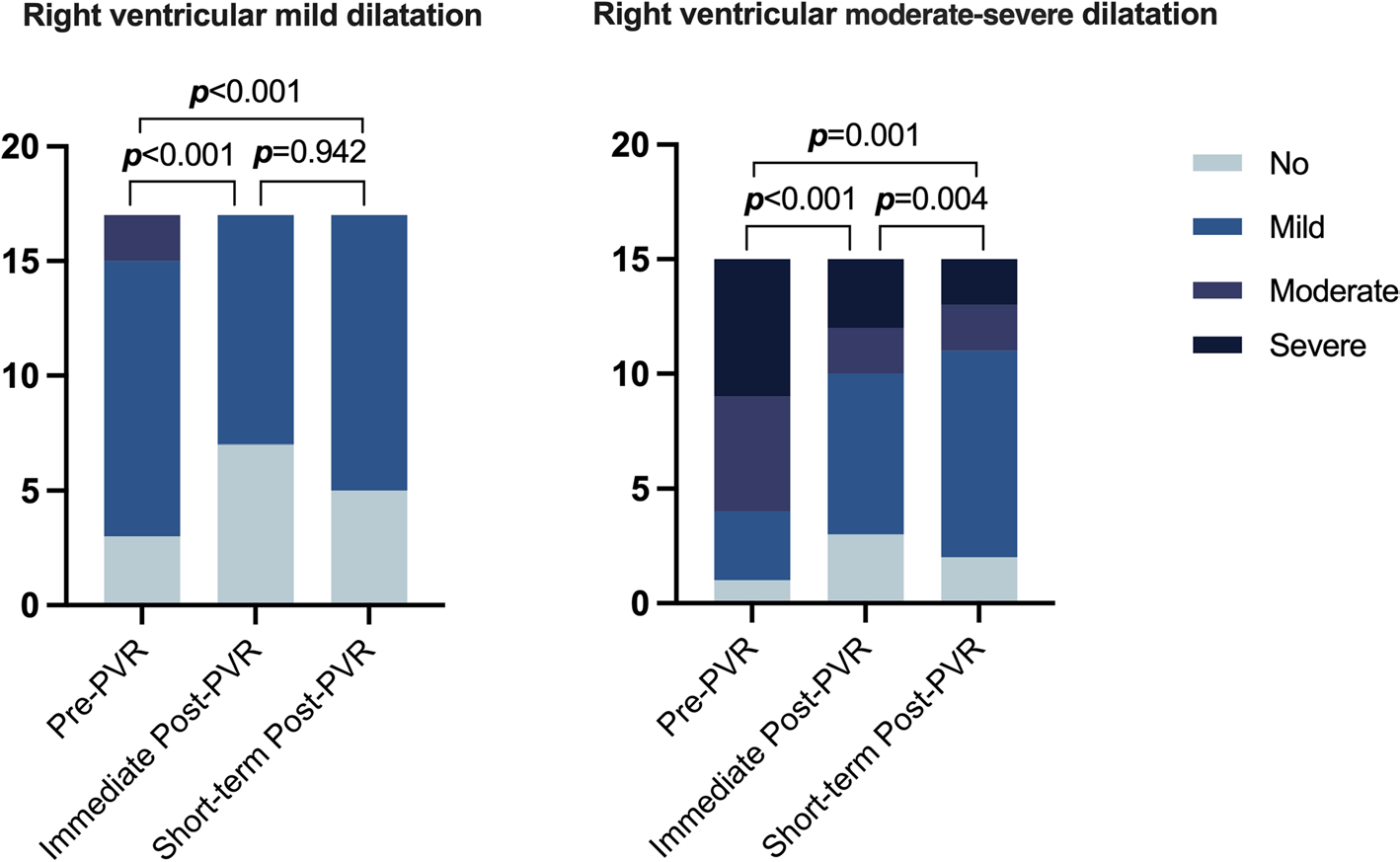
Proportion of tricuspid valve regurgitation degree of patients in different groups pre- and post-surgery

Among the 32 patients, the median follow-up time was 5 months. During this period, 3 out of 15 patients with RV moderate-severe dilatation experienced cardiovascular adverse events, such as paroxysmal chest tightness, frequent premature beats, and paroxysmal dyspnea. Meanwhile, 3 out of 17 patients with RV mild dilatation had adverse cardiovascular events, including chest pain with dizziness and acute myocardial ischemia. A comparison of early postoperative outcomes between the two groups showed that the RV mild dilatation group had a shorter postoperative hospital stay (6.59 [2.12] days versus 9.80 [5.10] days, *p* = 0.024) and a lower incidence of postoperative hypohepatia (0[0] % versus 4[26.7] %, *p* = 0.023) (Table S2). All patients underwent echocardiography at discharge and 3–6 months after surgery (Table 4 and Table 5). In the RV mild dilatation group, left atrial diameter, RVOT anteroposterior diameter index, RASID, early peak diastolic flow rate of tricuspid valve (E), and PR area improved immediately after the operation. However, there was no significant improvement during the short-term follow-up after discharge. Conversely, in the RV moderate-severe dilatation group, RVOT anteroposterior diameter index, E, doppler flow velocity of tricuspid annulus tissue during diastole (E’), and pulmonary valve velocity did not significantly improve immediately after surgery, but showed significant improvement during the short-term follow-up after discharge (Table 5). Moreover, TR improved immediately after surgery in the RV mild dilatation group and did not significantly change in the short-term follow-up after discharge. In contrast, patients in the RV moderate-severe dilatation group showed improvements both immediately after surgery and during the short-term follow-up after discharge (Fig. 3).

**Table 4:** Echocardiograph data of right ventricular mild dilatation group pre- and post-surgery (*n* = 17)

				*p* value		
	Pre-PVR	Immediate Post-PVR	Midterm Post-PVR	Pre- PVR vs. Immediate Post-PVR	Immediate Post- PVR vs Short-term Post-PVR	Pre- PVR vs Short-term Post-PVR
AO diameter, (mm)	29.65 (3.71)	28.88 (3.64)	29.59 (3.97)	0.458	0.563	0.951
LA diameter, (mm)	31.82 (2.92)	29.24 (4.01)	30.41 (5.37)	**0.001**	0.306	0.361
RVOT anteroposterior diameter index, (mm)	30.94 (5.38)	27.59 (4.06)	26.35 (4.43)	**0.011**	0.232	**0.002**
RASID, (mm)	52.88 (6.31)	49.83 (5.64)	50.65 (8.02)	**0.042**	0.619	0.237
RVSID, (mm)	60.29 (7.06)	59.41 (9.49)	57.24 (8.59)	0.705	0.239	0.128
MPA, (mm)	22.92 (3.24)	24.06 (3.03)	24.12 (3.04)	0.054	0.932	0.139
IVST, (mm)	9.01 (1.28)	8.91 (0.87)	8.72 (1.21)	0.741	0.486	0.361
E/e', (m/s)	10.59 (3.16)	11.82 (4.91)	12.00 (4.15)	0.213	0.912	0.273
E, (m/s)	0.58 (0.10)	0.73 (0.28)	0.64 (0.11)	**0.042**	0.22	0.07
E', (cm/s)	9.06 (2.47)	8.87 (2.58)	8.39 (2.63)	0.801	0.612	0.443
FAC, (%)	46.12 (5.81)	38.12 (8.55)	41.29 (2.31)	**0.002**	0.145	**0.004**
TAPSE, (mm)	16.97 (1.87)	15.41 (3.69)	15.21 (2.83)	0.109	0.822	0.052
S', (cm/s)	8.01 (1.24)	7.33 (1.63)	7.74 (1.59)	0.162	0.386	0.625
RVSP, (mmHg)	28.76 (13.90)	27.65 (10.19)	34.29 (10.96)	0.863	0.159	0.233
LVEF	66.41 (4.15)	65.59 (5.49)	65.06 (5.71)	0.625	0.818	0.465
TR, n (%)				** < 0.001**	0.942	** < 0.001**
no	3 (17.6)	7 (41.2)	5 (29.4)			
mild	12 (70.6)	10 (58.8)	12 (70.6)			
moderate	2 (11.8)	0 (0)	0 (0)			
severe	0 (0)	0 (0)	0 (0)			
PR area, (cm2)	11.24 (3.28)	1.00 (1.71)	1.25 (2.06)	** < 0.001**	0.697	** < 0.001**
PV peak gradient, (mmHg)	16.94 (15.71)	18.47 (11.02)	24.06 (13.89)	0.343	0.058	**0.028**
PV mean gradient, (mmHg)	16.41 (6.97)	\	10.82 (6.66)	\	\	**0.005**
PV velocity, (m/s)	1.96	1.91	1.84	0.709	0.804	0.54

Data are count (%), mean (SD), or median [25th–75th percentiles]; *p* values < 0.05 are shown in bold

*AO* Aod aortic, *LA* Left atrial, *RVOT* Right ventricular outflow tract, *RASID* Right atrial superior and inferior diameter, *RVSID* Right ventricular superior and inferior diameter, *MPA* Main pulmonary artery, *IVST* Interventricular septal thickness, *E/E*’ Early diastolic velocity of the tricuspid valve/early diastolic velocity of the lateral wall of the tricuspid annulus, *E* Early peak diastolic flow rate of tricuspid valve, *E* Doppler flow velocity of tricuspid annulus tissue during diastole, *FAC* Fractional area change, *TAPSE* Tricuspid annular plane systolic excursion, *S*’ Tissue Doppler tricuspid annulus systolic velocity, *RVSP* Right ventricular systolic pressure, *LVEF* Left ventricular ejection fraction, *TR* Tricuspid regurgitation, *PR* Pulmonary regurgitation, *PV* Pulmonary valve, *PS* Pulmonary stenosis

**Table 5:** Echocardiograph data of right ventricle moderate-severe dilatation group pre- and post-surgery (*n* = 15)

				*p* value		
	Pre-PVR	Immediate Post-PVR	Midterm Post-PVR	Pre- PVR vs. Immediate Post-PVR	Immediate Post- PVR vs Short-term Post-PVR	Pre- PVR vs Short-term Post-PVR
AO diameter, (mm)	28.87 (3.29)	28.33 (5.34)	29.00 (4.84)	0.458	0.563	0.951
LA diameter, (mm)	34.53 (7.21)	31.67 (7.95)	32.60 (7.21)	**0.001**	0.306	0.361
RVOT anteroposterior diameter index, (mm)	33.4 (7.52)	30.73 (9.87)	29.13 (8.58)	**0.011**	0.232	**0.002**
RASID, (mm)	63.07 (13.68)	54.93 (10.07)	54.47 (8.77)	**0.042**	0.619	0.237
RVSID, (mm)	67.00 (10.22)	67.93 (16.12)	65.47 (12.85)	0.705	0.239	0.128
MPA, (mm)	28.60 (5.84)	26.60 (4.45)	25.93 (4.38)	0.054	0.932	0.139
IVST, (mm)	9.03 (2.13)	9.46 (1.44)	9.15 (1.17)	0.741	0.486	0.361
E/e', (m/s)	10.60 (3.70)	13.93 (5.48)	14.53 (4.58)	0.213	0.912	0.273
E, (m/s)	0.56 (0.11)	0.65 (0.20)	0.69 (0.13)	**0.042**	0.22	0.07
E', (cm/s)	8.64 (2.44)	7.26 (2.34)	7.09 (2.11)	0.801	0.612	0.443
FAC, (%)	39.62 (10.22)	35.53 (7.75)	38.00 (3.93)	**0.002**	0.145	**0.004**
TAPSE, (mm)	17.23 (2.65)	14.01 (2.77)	14.62 (3.19)	0.109	0.822	0.052
S', (cm/s)	6.67 (1.28)	6.50 (1.42)	6.25 (1.46)	0.162	0.386	0.625
RVSP, (mmHg)	32.33 (9.15)	29.27 (19.05)	38.53 (14.11)	0.863	0.159	0.233
LVEF	60.53 (9.89)	60.27 (8.51)	64.07 (5.97)	0.625	0.818	0.465
TR, n (%)				** < 0.001**	0.942	** < 0.001**
no	1 (6.7)	3 (20.0)	2 (13.3%)			
mild	3 (20.0)	7 (46.7)	9 (60.0)			
moderate	5 (33.3)	2 (13.3)	2 (13.3)			
severe	6 (40.0)	3 (20.0)	2(13.3)			
PR area, (cm2)	10.59 (2.92)	1.69 (2.51)	0.73 (1.84)	** < 0.001**	0.204	** < 0.001**
PV peak gradient, (mmHg)	11.29 (7.75)	14.72 (6.79)	18.13 (9.35)	0.084	0.244	0.065
PV mean gradient, (mmHg)	14.67 (8.23)		12.40 (8.41)			0.544
PV velocity, (m/s)	1.55 (0.64)	1.65 (0.63)	2.1 (0.62)	0.525	**0.027**	**0.023**

Data are count (%), mean (SD), or median [25th–75th percentiles], p values less than 0.05 are shown in bold

*AO* Aod aortic, *LA* Left atrial, *RVOT* Right ventricular outflow tract, *RASID* Right atrial superior and inferior diameter, *RVSID* Right ventricular superior and inferior diameter, *MPA* Main pulmonary artery, *IVST* Interventricular septal thickness, *E/E*’, Early diastolic velocity of the tricuspid valve/early diastolic velocity of the lateral wall of the tricuspid annulus, *E* Early peak diastolic flow rate of tricuspid valve, *E*’ Doppler flow velocity of tricuspid annulus tissue during diastole, *FAC* Fractional area change, *TAPSE* Tricuspid annular plane systolic excursion, *S*’ Tissue Doppler tricuspid annulus systolic velocity, *RVSP* Right ventricular systolic pressure, *LVEF* Left ventricular ejection fraction, *TR* Tricuspid regurgitation, *PR* Pulmonary regurgitation, *PV* Pulmonary valve, *PS* Pulmonary stenosis

### RV dilatation score

**Fig. 4:**
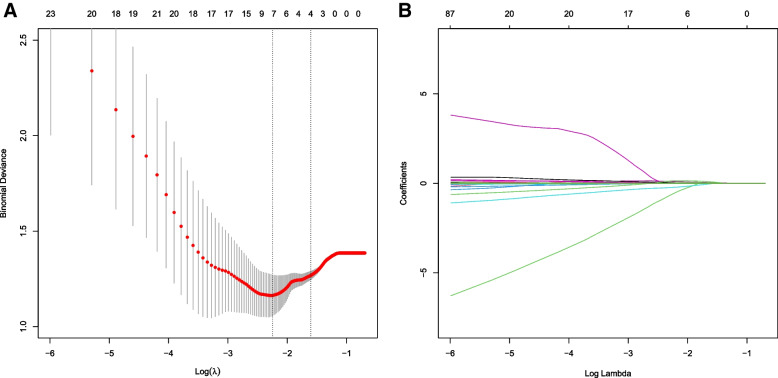
Clinical variable selection using the LASSO regression model. **A** The y-axis indicates the binomial deviance, while the lower x-axis indicates the log (lambda) and the upper x-axis represents the average number of predictors. Dotted vertical lines were drawn at the optimal values using the minimum criteria and 1 standard error of the minimum criteria. The tuning parameter (λ) was selected in the LASSO model via fivefold cross-validation based on minimum criteria. **B** The y-axis indicates the LASSO coefficient, while the lower x-axis indicates the log (lambda) and the upper x-axis represents the average number of predictors

**Fig. 5:**
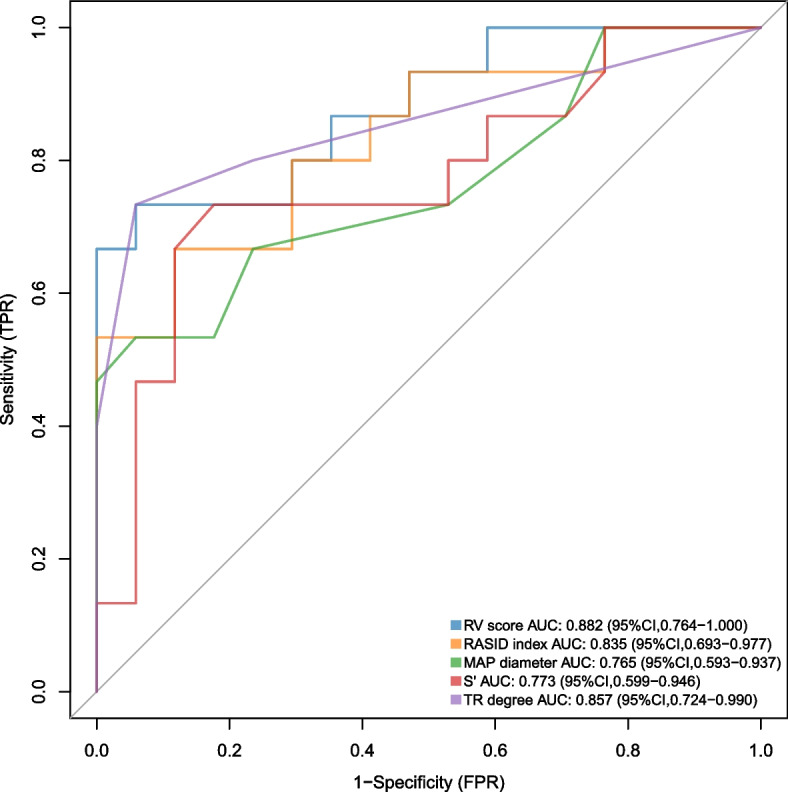
Receiver operator characteristic curve

Through univariate analysis and correlation analysis, we obtained variables that had significant differences between the two groups of patients and were significantly correlated with CMR data. To construct a scoring model for RV dilatation, we used LASSO regression analysis to select the most significant variables from those variables, with grouping factors as the dependent variables (Fig. 4). When lambda = 0.034, four variables with non-zero coefficients were selected and the RV dilatation score was calculated according to the weights of these four variables: *RV dilatation score* = 0.045*RASID index + 0.025*MPA diameter—0.022*S' + 0.082*TR degree (with 1, 2, 3, and 4 representing no, mild, moderate, and severe TR, respectively). To evaluate the performance of the scoring model, a ROC curve was plotted, with sensitivity as the ordinate and (1- specificity) as the abscissa. The area under ROC curve (AUC) of the RV dilatation score was 0.882 (95%CI: 0.764–1.000), with a sensitivity of 94.1% and a specificity of 73.3%, and the optimal cut-off value was 2.35. The ROC curve of each variable in the scoring model is summarized in Table S3 and Fig. 5. The results of calibration curve also show that the scoring model has good consistency (Figure S3). The score of all rTOF patients included in our study was caculated by this formula, and the score were correlated with RVESVI (*r* = 0.53, *p* < 0.01) and RVEDVI (*r* = 0.55, *p* < 0.01) (Figure S4).

## Discussion

### Preoperative clinical features

With advancements in PVR technology, it is widely accepted to perform PVR before irreversible RV dilatation and heart function deterioration occurs in patients with rTOF [9–11]. Previous studies have shown that the cumulative incidence of long-term reoperation in patients with rTOF is 44%, with a median of 24 years after the initial repair [12, 13]. In a longitudinal cohort of 144 rTOF patients, Cuypers et al. reported a cumulative incidence of 40% of PVR over 35 years [12]. Our study cohort showed that patients receiving PVR had a median age of 25 years and had undergone the primary surgery 16.5 years ago. These findings suggest that with the progression of technology and treatment concepts, more patients with rTOF receive PVR treatment earlier.

Cardiac electrophysiology is an important evaluation method for patients with rTOF, and the presence of persistent arrhythmias before surgery often indicates a poor prognosis [14–16]. Multiple studies have shown that QRS duration is closely related to RV dilatation [14]. Guidelines from the European Society of Cardiology suggest that QRS duration > 180mms be used as an indication of PVR [14, 17]. However, in our research cohort, there was no significant difference between groups in QRS duration, even in the RV moderate-severe dilatation group, the median QRS duration was still less than 180mms. This paradoxical result requires further explored with larger sample sizes.

While laboratory tests are not routinely used to assess patients with rTOF, some sensitive biomarkers reflecting right ventricular dysfunction play an auxiliary role in risk stratification [18]. Hirono et al. found that NT-proBNP levels were significantly higher in patients with PVR indication than in patients without PVR indication and that NT-proBNP was an independent predictor of PVR in patients with rTOF [19]. A prospective cohort study by Bleck et al. found that NT-proBNP was an independent predictor of adverse events in patients with rTOF, with an AUC of 0.873. The critical value of 168 ng/L was used to predict the sensitivity and specificity of adverse events, which were 84.6% and 75%, respectively [20]. Our study cohort also showed that NT-proBNP is a valuable indicator. Surprisingly, we found significant differences in TP and LDH levels between the two groups. Some studies have found that concentrations of certain proteins related to liver fibrosis in peripheral blood increase significantly [21]. Considering the influence of right heart pressure on liver function, the differences in peripheral blood protein levels could be partially explained.

### Preoperative CMR and echocardiography

rTOF patients are characterized by RV dilatation and dysfunction. Consequently, CMR and echocardiography have been studied extensively to aid in the evaluation of these patients. Due to the unique anatomy of the RV, CMR is the preferred imaging modality for evaluating the right heart [22, 23] and numerous studies have investigated CMR measurements of RV size to determine the optimal surgical indication for PVR [24–27]. Guidelines recommend RVEDVi > 160 mL/m2 or RVESVi > 80 mL/m2 as the optimal indications for asymptomatic rTOF patients [7, 17, 28]. Although echocardiography has limitations in quantitatively assessing RV size and pulmonary regurgitation [29], recent advancements in techniques and further clinical studies have shown promising results by correlating echocardiography parameters with CMR, suggesting its potential use as a cost-effective screening method or as a complement to CMR [30].

In correlation analysis, we observed a significant correlation between LVEF measured by echocardiography and RVEDVI, RVESVI, and RVEF in patients with rTOF. This finding supports the concept of left and right ventricular interactions in rTOF patients, which has been reported in other studies [24, 31].

We found a significant difference in the RASID index between groups of patients with rTOF, which was also significantly correlated with CMR parameters. Previous studies have shown that RASID index is dilated in rTOF patients and decreases after PVR [26]. Furthermore, RASID has been demonstrated to reflect RV diastolic function and to be a predictor of adverse cardiovascular events after PVR [32]. RA dilatation has also been suggested to affect right heart remodeling and to predict atrial arrhythmia, particularly in patients with rTOF [33]. In patients with rTOF, MPA is often larger than in normal subjects [34]. In our study cohort, MPA diameter in patients with RV moderate-severe dilatation was larger than that in patients with RV mild dilatation. Other studies paid more attention to hemodynamic factors in pulmonary artery, with some suggesting that increased longitudinal circulation of MPA was associated with RV dysfunction [35]. In addition, it has been shown that end-diastolic anterior blood flow in the MPA is associated with poor outcomes in patients with rTOF [36]. We also observed that LPA diameter was significantly larger in RV moderate-severe dilatation patients while the right pulmonary artery (RPA) was not significantly different. The anatomical differences between the LPA and RPA may contribute to this finding, but further research is needed to confirm this hypothesis. Tissue Doppler tricuspid annulus systolic velocity(S’) can reflect the overall systolic function of the ventricle [37]. Patients with rTOF often exhibit decreased diastolic function during the compensatory period of right ventricular dysfunction, which then progress into a decompensated period characterized by decreased systolic function [38]. In our cohort, overall S’ decreased, and S’ was significantly lower in the RV dilatation group than in the RV mild dilatation group. This indicated that rTOF patients had right ventricular dysfunction and that the RV moderate-severe dilatation group was more severe. These findings indicate that rTOF patients have impaired RV function and that the group with RV moderate-severe dilatation may more severe.

### RV dilatation score

Finally, four echocardiography parameters, RASID index, S’, MAP diameter, and TR degree, were selected to comprehensively determine the severity of right ventricular dilatation in our study. There were significant differences in these four parameters between the two groups. Moreover, they were significantly correlated with CMR data (RVESVI and RVEDVI), respectively. This score has a high sensitivity (94.1%) and a relatively low specificity (77.3%), so this score is suitable as a screening indicator. A rTOF patient with moderate to severe PR needs only a simple echocardiography to calculate the RV dilatation score. The relationship between this score and the cut-off value can then be used to screen for patients who need further treatment. It is important to emphasize that RV dilatation score is not a substitute for CMR. When a patient's RV dilatation score > 2.35, it indicates that the patient needs to be considered for PVR intervention, and the patient still needs to undergo more precise diagnostic tests including CMR before this can be done. In a word, the clinical significance of this score is to screen out patients who need intervention through echocardiography, which is a relatively simple and inexpensive method, to avoid delayed treatment of these patients.

### Short-term outcome

Several large-sample cohort studies have concluded that patients with rTOF with RV dilatation have a significantly increased incidence of long term cardiovascular adverse events. The threshold of RV dilatation determined based on these studies was RVEDVI ≥ 160 ml/m2 or RVESVI ≥ 80 ml/m2, which was also the basis for grouping patients in our study [2, 21, 39]. We conducted short-term postoperative follow-up of rTOF patients and found that few patients in both groups experienced postoperative cardiovascular adverse events. However, it is worth noting that patients in the RV moderate-severe group had a significantly higher incidence of early postoperative liver insufficiency and a longer postoperative hospital stay. We consider that the occurrence of liver insufficiency is mainly related to preoperative RV dysfunction. When comparing echocardiography data at different time periods, we found that RV size and function of rTOF patients recovered over time after PVR surgery, which was consistent with the study of Heng et al. [26]. However, the recovery of RV function over time varied between the two groups, some echocardiography parameters reflecting right heart function in RV moderate-severe dilatation group patients were continuously improved immediately after surgery and 3–6 months after surgery, whereas in the RV mild dilatation group, there were only significant improvements immediately after surgery, with no significant changes at 3–6 months after surgery. These findings suggest that patients with RV moderate-severe dilatation have a poorer prognosis and require a longer recovery time to RV function.

The different PVR approaches affect the short-term prognosis of patients to some extent. A more reasonable research programme would be to conduct subgroup analysis based on surgical approaches or to focus on patients' long-term prognosis (surgical approaches has less effect on long-term prognosis). However, due to the limitations of the study, we did not have enough cases to conduct a subgroup analysis. These results need to be treated with caution.

### Limitation and prospects

There are several limitations to our study, including the small sample size, short follow-up time, and limited number of patients with postoperative adverse cardiovascular events. Additionally, due to variations in the anatomy of the pulmonary artery, patients may receive different surgical interventions during the initial operation for tetralogy of Fallot, which can lead to differences in postoperative hemodynamics. Unfortunately, the data of our cohort was lost due to the patients undergoing tetralogy of Fallot surgery in the distant past.

Our study is a preliminary exploratory work, as PVR timing is a complex issue to balance (1) preoperative risk; (2) Short-term and long-term ventricular function gains after surgery; (3) Persistence of operative effectiveness. To further explore it requires a larger sample size and longer follow-up time, and ultimately helping us to improve our understanding of how to treat patients with rTOF.

## Conclusion

In summary, echocardiography can serve as a reliable screening method for assessing RV size in rTOF patients. The RV dilatation score derived from echocardiography can effectively reflect the extent of right ventricular dilatation within a specific range. Within a range, higher scores suggest larger right ventricles and poorer cardiac function, and are associated with no short-term improvement in postoperative outcomes. Notably, when the RV dilatation score exceeds 2.35, it suggests that patients require further diagnosis and treatment.

### Supplementary Information

**Additional file 1: Figure S1. **Correlation analysis heat map. Blue is positive correlation; red is negative correlation. The depth of color represents the strength of correlation. "*", "**" and "***" respectively represent P value less than 0.05, 0.01 and 0.001. TAPSE, tricuspid annular plane systolic excursion; RVEF, right ventricular ejection fraction; FAC, fractional area change; S, tissue doppler tricuspid annulus systolic velocity; LVEF, left ventricular ejection fraction; AO, aod aortic; TR, tricuspid regurgitation; LPA, left pulmonary artery; LVDD, left ventricular diastolic diameter; RA, right atrial superior and inferior diameter; RV, right ventricular superior and inferior diameter; MPA, main pulmonary artery; RVEDVI, right ventricular end-diastolic volume index; RVESVI, right ventricular end-systolic volume index.

**Additional file 2: Figure S2.**Linear correlation analysis between different echocardiographic parameters and RVEF. RVEF, right ventricular ejection fraction; RASID, right atrial superior and inferior diameter; FAC, fractional area change; S’, tissue doppler tricuspid annulus systolic velocity; LVEF, left ventricular ejection fraction; LVDD, left ventricular diastolic diameter; RVSID, right ventricular superior and inferior diameter.

**Additional file 3: Figure S3. **Calibration curve.

**Additional file 4: Figure S4. **Linear correlation analysis between CMR data and RV dilatation score. RVEDVI, right ventricular end-diastolic volume index; RVESVI, right ventricular end-systolic volume index.

**Additional file 5: Table S1. **Correlation analysis of echocardiography data and cardiac magnetic resonance data.

**Additional file 6: Table S2. **Early postoperative outcome.

**Additional file 7: Table S3. **ROC curve parameters of different variables.

## Data Availability

Study data can be obtained by sending a request to the corresponding author.

## Abbreviations

rTOF: Repaired tetralogy of Fallot
RV: Right ventricular
CMR: Cardiac magnetic resonance
RVEDVI: Right ventricular end-diastolic volume index
RVESVI: Right ventricular end-systolic volume index
PR: Pulmonary regurgitation
PVR: Pulmonary valve replacement
RVEF: Right ventricular ejection fraction
CMR-LVEF: CMR-derived left ventricular ejection fraction
LVCO: Left ventricular cardiac output
RVCO: Right ventricular cardiac output
TR: Tricuspid regurgitation
LDH: Lactate dehydrogenase
N-BNP: N-terminal B-type natriuretic peptide
TP: Total protein
RASID: Right atrial superior and inferior diameter
S’: Tissue doppler tricuspid annulus systolic velocity
MPA: Main pulmonary artery
LPA: Left pulmonary artery
RPA: Right pulmonary artery
LVDD: Left ventricular diastolic diameter
