# Supplementary figures and images for: Right ventricular dilatation score: a new assessment to right ventricular dilatation in adult patients with repaired tetralogy of Fallot
Source: BMC Cardiovasc Disord. 2023 Sep 14;23:458. doi: 10.1186/s12872-023-03487-2 (PMC10500856; doi:10.1186/s12872-023-03487-2)

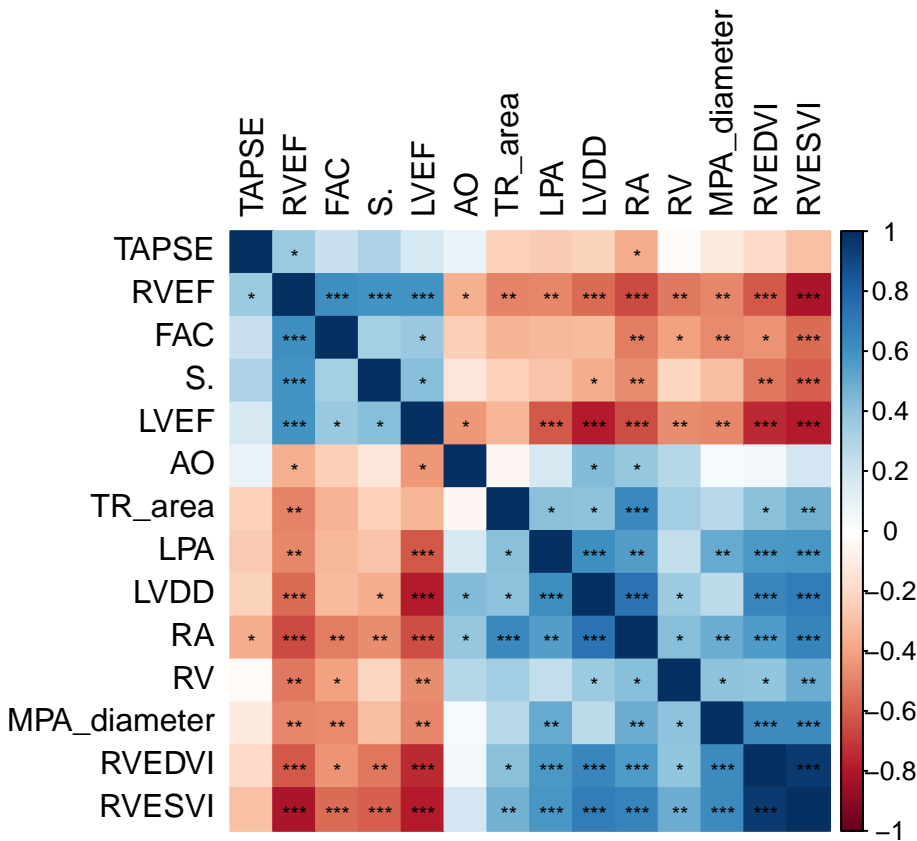

Supplement: Supplementary file 1 — Additional file 1: Figure S1. Correlation analysis heat map. Blue is positive correlation; red is negative correlation. The depth of color represents the strength of correlation. "*", "**" and "***" respectively represent P value less than 0.05, 0.01 and 0.001. TAPSE, tricuspid annular plane systolic excursion; RVEF, right ventricular ejection fraction; FAC, fractional area change; S, tissue doppler tricuspid annulus systolic velocity; LVEF, left ventricular ejection fraction; AO, aod aortic; TR, tricuspid regurgitation; LPA, left pulmonary artery; LVDD, left ventricular diastolic diameter; RA, right atrial superior and inferior diameter; RV, right ventricular superior and inferior diameter; MPA, main pulmonary artery; RVEDVI, right ventricular end-diastolic volume index; RVESVI, right ventricular end-systolic volume index. [file 12872_2023_3487_MOESM1_ESM.pdf]

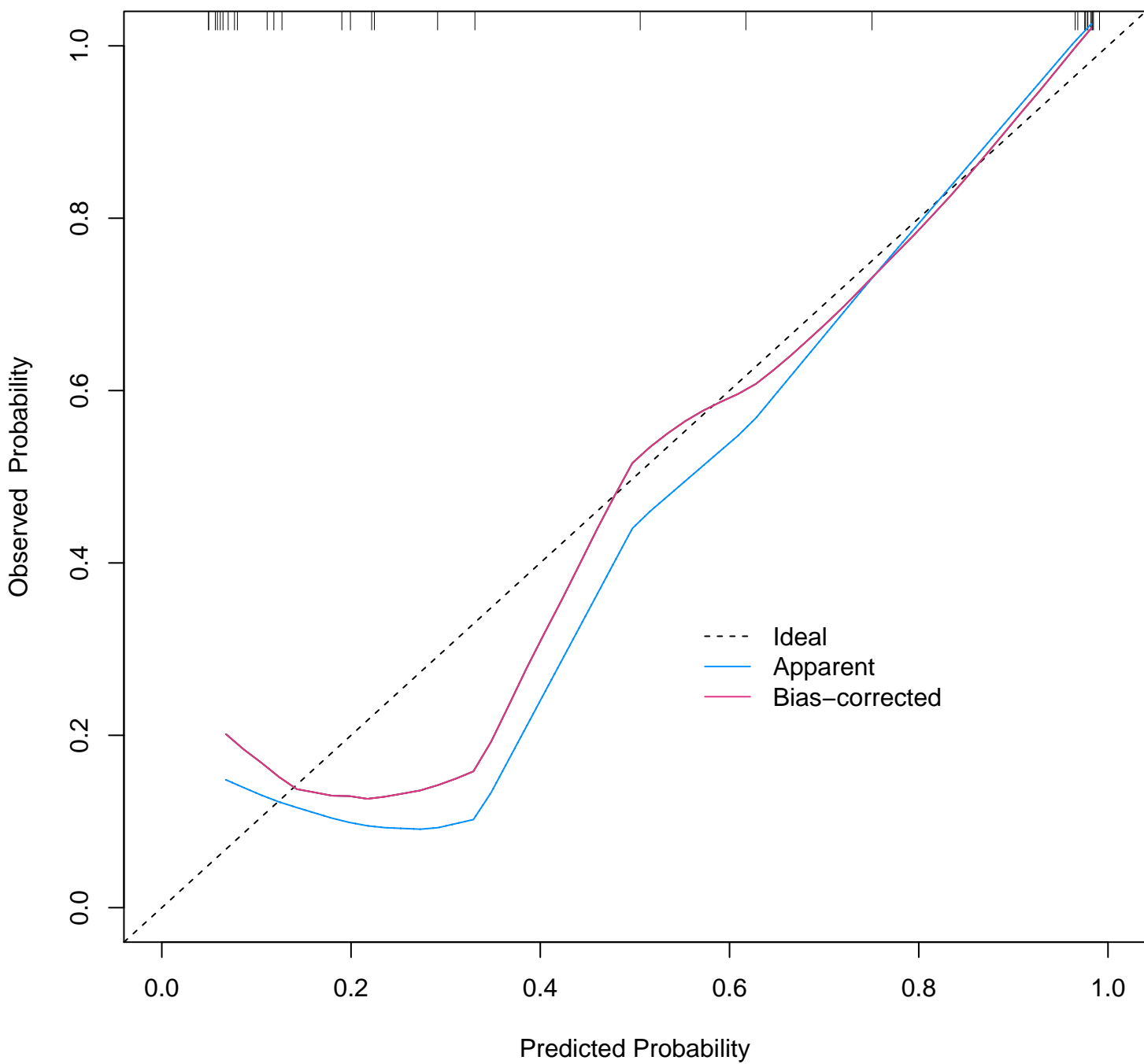

Supplement: Supplementary file 3 — Additional file 3: Figure S3. Calibration curve. [file 12872_2023_3487_MOESM3_ESM.pdf]

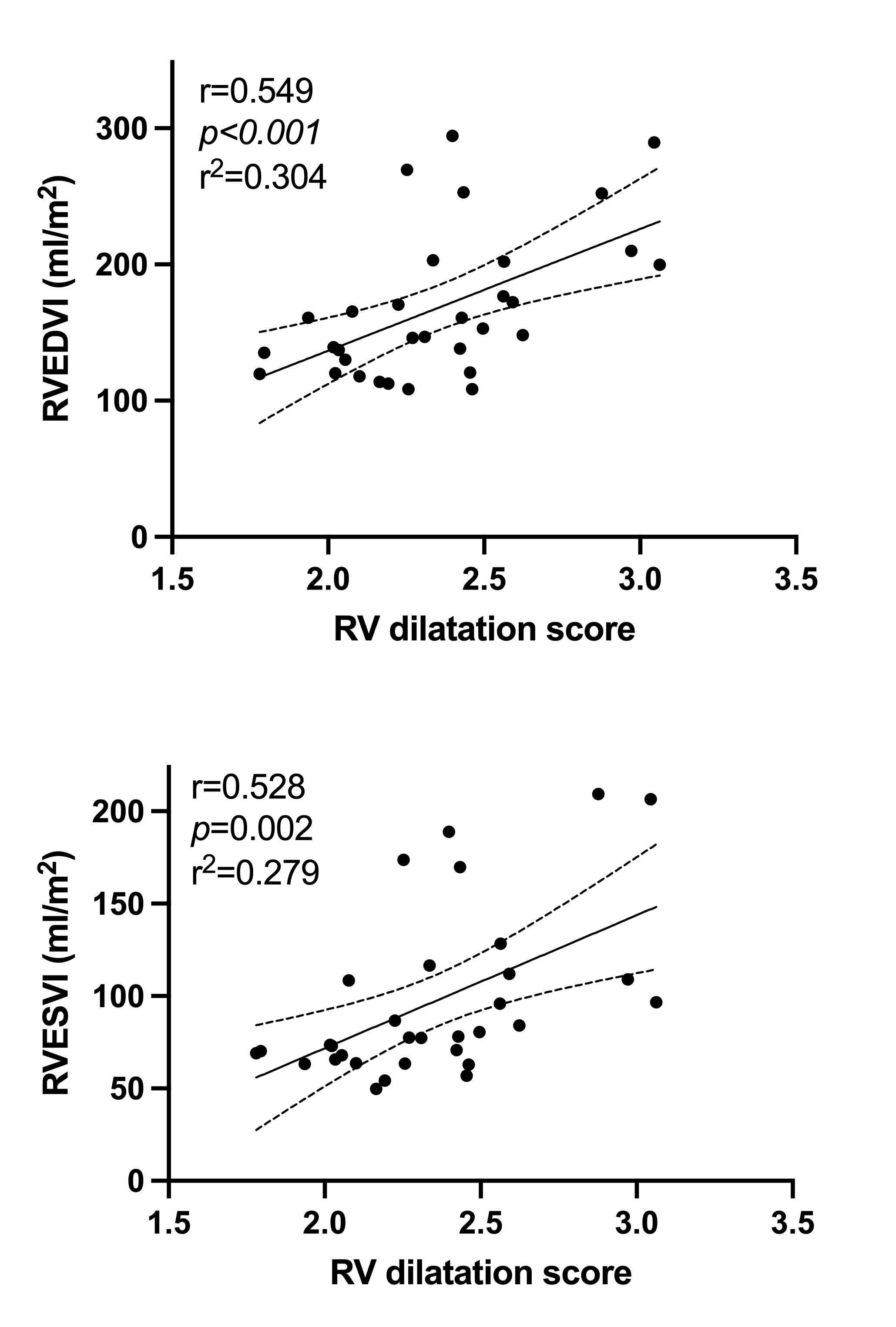

Supplement: Supplementary file 4 — Additional file 4: Figure S4. Linear correlation analysis between CMR data and RV dilatation score. RVEDVI, right ventricular end-diastolic volume index; RVESVI, right ventricular end-systolic volume index. [file 12872_2023_3487_MOESM4_ESM.tiff]
