# Supplementary material for: Right ventricular dilatation score: a new assessment to right ventricular dilatation in adult patients with repaired tetralogy of Fallot
Source: BMC Cardiovasc Disord. 2023 Sep 14;23:458. doi: 10.1186/s12872-023-03487-2 (PMC10500856; doi:10.1186/s12872-023-03487-2)
Supplement: Supplementary file 5 — Additional file 5: Table S1. Correlation analysis of echocardiography data and cardiac magnetic resonance data. [file 12872_2023_3487_MOESM5_ESM.docx]

| **Table S1** Correlation analysis of echocardiography data and cardiac magnetic resonance data | | | | | | | | |
| --- | --- | --- | --- | --- | --- | --- | --- | --- |
|  | RVEDVI | |  | RVESVI | |  | RVEF | |
|  | r | *p* |  | r | *p* |  | r | *p* |
| AO | 0.06 | 0.747 |  | 0.18 | 0.312 |  | -0.36 | 0.046 |
| LA | 0.34 | 0.059 |  | 0.44 | 0.011 |  | -0.48 | 0.005 |
| RASID | 0.57 | <0.001 |  | 0.66 | <0.001 |  | -0.65 | <0.001 |
| RVSID | 0.39 | 0.026 |  | 0.50 | 0.004 |  | -0.53 | 0.002 |
| LVDD | 0.65 | <0.001 |  | 0.70 | <0.001 |  | -0.57 | <0.001 |
| MPA diameter | 0.63 | <0.001 |  | 0.64 | <0.001 |  | -0.48 | 0.005 |
| LPA | 0.57 | <0.001 |  | 0.59 | <0.001 |  | -0.48 | 0.005 |
| FAC | -0.44 | 0.011 |  | -0.56 | <0.001 |  | 0.61 | <0.001 |
| TAPSE | -0.20 | 0.274 |  | -0.29 | 0.103 |  | 0.37 | 0.038 |
| S' | -0.52 | 0.002 |  | -0.60 | <0.001 |  | 0.59 | <0.001 |
| LVEF | -0.75 | <0.001 |  | -0.79 | <0.001 |  | 0.59 | <0.001 |
| TR area | 0.42 | 0.018 |  | 0.47 | <0.001 |  | -0.49 | 0.004 |
|  |  |  |  |  |  |  |  |  |
| RVEDVI, right ventricular end-diastolic volume index; RVESVI, right ventricular end-systolic volume index; RVEF, right ventricular ejection fraction; AO, aod aortic; RASID, right atrial superior and inferior diameter; RVSID, right ventricular superior and inferior diameter; LVDD, left ventricular diastolic diameter; MPA, main pulmonary artery; LPA, left pulmonary artery; FAC, fractional area change; TAPSE, tricuspid annular plane systolic excursion; S', tissue Doppler tricuspid annulus systolic velocity; LVEF, left ventricular ejection fraction; TR, tricuspid regurgitation. | | | | | | | | |
