# Supplementary material for: Right ventricular dilatation score: a new assessment to right ventricular dilatation in adult patients with repaired tetralogy of Fallot
Source: BMC Cardiovasc Disord. 2023 Sep 14;23:458. doi: 10.1186/s12872-023-03487-2 (PMC10500856; doi:10.1186/s12872-023-03487-2)
Supplement: Supplementary file 6 — Additional file 6: Table S2. Early postoperative outcome. [file 12872_2023_3487_MOESM6_ESM.docx]

| **Table S2** Early postoperative outcome | | | | |
| --- | --- | --- | --- | --- |
|  | Overall | Right ventricular mild dilatation | Right ventricular moderate-sever dilatation | *p* |
| Post hospital stay (day) | 8.09 (4.09) | 6.59 (2.12) | 9.80 (5.10) | **0.024** |
| Intubation time (min) | 382.50 [176.25, 845.25] | 360.00 [180.00, 595.00] | 429.00 [227.50, 1130.00] | 0.355 |
| ICU time (h) | 28.50 [20.23, 67.88] | 23.00 [20.25, 44.00] | 43.00 [21.21, 93.25] | 0.335 |
| Drainage 24 hours after operation(ml) | 147.50 [51.25, 282.50] | 100.00 [10.00, 220.00] | 215.00 [97.50, 355.00] | 0.191 |
| Post pulmonary infection (%) | 2 (6.2) | 1 (5.9) | 1 (6.7) | 0.927 |
| Post infraction (%) | 1 (3.1) | 1 (5.9) | 0 (0.0) | 0.34 |
| Post arrhythmia (%) | 1 (3.1) | 1 (5.9) | 0 (0.0) | 0.34 |
| Post hypohepatia (%) | 4 (12.5) | 0 (0.0) | 4 (26.7) | **0.023** |
| Post chylothorax (%) | 2 (6.2) | 0 (0.0) | 2 (13.3) | 0.12 |

Data are count (%), mean (SD), or median [25th–75th percentiles], p values less than 0.05 are shown in bold.
