# Supplementary material for: Right ventricular dilatation score: a new assessment to right ventricular dilatation in adult patients with repaired tetralogy of Fallot
Source: BMC Cardiovasc Disord. 2023 Sep 14;23:458. doi: 10.1186/s12872-023-03487-2 (PMC10500856; doi:10.1186/s12872-023-03487-2)
Supplement: Supplementary file 7 — Additional file 7: Table S3. ROC curve parameters of different variables. [file 12872_2023_3487_MOESM7_ESM.docx]

| **Table S3** ROC curve parameters of different variables | | | |
| --- | --- | --- | --- |
| variables | AUC（95%CI） | specificity | sensitivity |
| RV-dilatation-score | 0.898(0.772-1.000) | 0.667 | 1 |
| RASID index | 0.835(0.693-0.977) | 0.667 | 0.882 |
| TR degree | 0.804(0.646-0.962) | 0.882 | 0.733 |
| MPA diameter | 0.765(0.593-0.937) | 0.941 | 0.533 |
| S' | 0.773(0.599-0.946) | 0.733 | 0.824 |

RVSID, right ventricular superior and inferior diameter; TR, tricuspid regurgitation; MPA, main pulmonary artery; S', tissue Doppler tricuspid annulus systolic velocity.
